# Applying assisted reproductive technology and reproductive management to reduce CO_2_-equivalent emission in dairy and beef cattle: a review

**DOI:** 10.1590/1984-3143-AR2023-0060

**Published:** 2023-09-08

**Authors:** Pietro Sampaio Baruselli, Laís Ângelo de Abreu, Vanessa Romário de Paula, Bruno Carvalho, Emanuelle Almeida Gricio, Fernando Kenji Mori, Lígia Mattos Rebeis, Sofía Albertini, Alexandre Henrily de Souza, Michael D’Occhio

**Affiliations:** 1 Departamento de Reprodução Animal, Faculdade de Medicina Veterinária e Zootecnia, Universidade de São Paulo, São Paulo, SP, Brasil; 2 Instituto Paulista de Ensino e Pesquisa, Empresa Brasileira de Pesquisa Agropecuária – EMBRAPA, Juiz de Fora, MG, Brasil; 3 Cargill Nutrição Animal, Campinas, SP, Brasil; 4 School of Life and Environmental Sciences, Faculty of Science, The University of Sydney, Sydney, Australia

**Keywords:** cattle, enteric methane, efficiency, fertility, assisted reproductive technology

## Abstract

Methane emission from beef and dairy cattle combined contributes around 4.5-5.0% of total anthropogenic global methane. In addition to enteric methane (CH_4_) produced by the rumen, cattle production also contributes carbon dioxide (CO_2_) (feed), nitrous oxide (N_2_O) (feed production, manure) and other CH_4_ (manure) to the total greenhouse gas (GHG) budget of beef and dairy production systems. The relative contribution in standard dairy systems is typically enteric CH_4_ 58%, feed 29% and manure 10%. Herds with low production efficiency can have an enteric CH_4_ contribution up to 90%. Digestibility of feed can impact CH_4_ emission intensity. Low fertility herds also have a greater enteric CH_4_ contribution. Animals with good feed conversion efficiency have a lower emission intensity of CH_4_/kg of meat or milk. Feed efficient heifers tend to be lean and have delayed puberty. Fertility is a major driver of profit in both beef and dairy cattle, and it is highly important to apply multi-trait selection when shifting herds towards improved efficiency and reduced CH_4_. Single nucleotide polymorphisms (SNPs) have been identified for feed efficiency in cattle and are used in genomic selection. SNPs can be utilized in artificial insemination and embryo transfer to increase the proportion of cattle that have the attributes of efficiency, fertility and reduced enteric CH_4_. Prepubertal heifers genomically selected for favourable traits can have oocytes recovered to produce IVF embryos. Reproductive technology is predicted to be increasingly adopted to reduce generation interval and accelerate the rate of genetic gain for efficiency, fertility and low CH_4_ in cattle. The relatively high contribution of cattle to anthropogenic global methane has focussed attention on strategies to reduce enteric CH_4_ without compromising efficiency and fertility. Assisted reproductive technology has an important role in achieving the goal of multiplying and distributing cattle that have good efficiency, fertility and low CH_4_.

## Introduction

The global population of beef and dairy cattle combined is approximately 1.5 billion. Amongst domestic herbivores globally, cattle contribute about 20% of meat and 85% of milk. The global demand for meat and milk is projected to increase by 57% and 48%, respectively, between 2005 and 2050 (Alexandratos and Bruinsma, 2012). Cattle, therefore, will continue to have a very important role in future global food security (Davis and White, 2020). Beef and dairy production occur across diverse environments and in both extensive and intensive systems (Faverdin et al., 2022). Extensive cattle grazing is found in rangelands and savannas that are suited to low-input and low-cost animal production. Intensive beef and dairy systems utilize, and add value to, feed sources that are either unsuitable or surplus to human consumption. Grazing lands cover about 25% of the global landmass (Mottet et al., 2018) and intensive beef accounts for <15% of global beef production (Mottet et al., 2017). The environmental footprint of cattle production has received increased attention globally (Knapp et al., 2014; Faverdin et al., 2022). Methane emission from cattle has been recognised for around 30 years (Johnson and Johnson, 1995) and has become a particular focus as cattle contribute around 4.5-5.0% of total anthropogenic global methane (Wallace et al., 2015; Hayes et al., 2016; de Haas et al., 2021; Faverdin et al., 2022; Hossein-Zadeh, 2022; Galyean and Hales, 2023). Most methane produced by cattle is from enteric fermentation of complex carbohydrates into simple sugars by methanogenic protozoa (Bowen et al., 2020). The biology and function of the rumen has been well reviewed (Ross et al., 2013; Knapp et al., 2014). The ability to digest cellulolytic material into usable energy and protein is arguably the greatest advantage but also the greatest disadvantage of cattle. The relative abundance of ruminal methanogenic and non-methanogenic microbes influence the amount of methane produced by an individual animal (Bowen et al., 2020). The population of ruminal microbes can now be determined by microbial gene abundance (Roehe et al., 2016).

Assisted reproductive technologies can have a major impact on improving productivity in beef and dairy cattle. Artificial insemination (AI) and multiple ovulation and embryo transfer (MOET) increase the rate of dissemination of animals with traits that have high genetic merit and high productive capacity. However, the mature technologies of AI and MOET do not increase the rate of genetic gain from one generation to the next. The latter is controlled by generation interval which is relatively long in cattle (Schefers and Weigel, 2012; Kasinathan et al. 2015). Generation interval can be shortened in cattle by utilising oocytes from heifers early in life to produce IVF embryos (Baruselli et al., 2016; Baldassarre and Bordignon, 2018). This review seeks to demonstrate how assisted reproductive technology (ART) and reproductive management can be used to generate cattle that have improved efficiency and produce less methane.

## Reproductive efficiency in cattle and application of artificial insemination to improve efficiency and reduce methane emission

In beef cattle, the cow-calf unit utilizes approximately 70% of resources. Selection for reproductive efficiency therefore has a major bearing on both efficiency and profitability. With high reproductive efficiency, fewer cows are required to produce the next generation of calves, and this reduces resource requirement, herd methane production, and costs (Hegarty and McEwan, 2010). Also, reproductively inefficient cows are removed from herds. In a United States beef production system, an improvement in reproductive efficiency (0.5 to 1 calves/year) resulted in a 34% reduction in water use, 44% reduction in land use, and 39% reduction in the CO_2_-equivalent (CO_2_-eq) footprint (Davis and White, 2020). ART can be incorporated into beef breeding programs to further improve efficiency and reduce CO_2_-eq emission intensity. In Brazil, the use of timed artificial insemination (TAI) in a breeding herd reduced age at first calving from 48 to 24 months and increased weaning rate from 60% to 80% compared with natural mating (Abreu et al., 2022). There was a 37.7% reduction in pasture required and 85.4% reduction in CO_2_-eq to produce 400 calves (Abreu et al., 2022). The CO_2_-eq was calculated according to livestock units (1 LU=450 kg of live weight) and a stocking rate of 1 LU per hectare of pasture was estimated to produce calves (Figueiredo et al., 2017). The low reproductive efficiency system (natural mating) emitted 3,714.5 tons of CO_2_-eq per year while the high reproductive efficiency system (TAI) emitted 2,311.3 tons of CO_2_-eq annually. The TAI system generated US$84,196 in credit for reducing CO_2_-eq emissions (quoted at US$60 per 1-ton CO_2_-eq). TAI has been applied in beef heifers to reduce age at first pregnancy and calving (Baruselli et al., 2017) which impacts lifetime reproductive efficiency and CO_2_-eq emissions. TAI can also be utilized to manage inter-calving intervals so that cows produce a calf annually (Sá et al., 2013; Baruselli et al., 2018a).

The same basic principles addressed above apply in dairy cattle (Hutchinson et al., 2013). For example, lowering the age at first calving and culling frequency reduced the number of replacement heifers needed and enteric methane emission per unit of kg energy-corrected milk (CH_4_/ECM; Knapp et al., 2014). Improving the fertility of dairy herds can potentially reduce methane emission by up to 25% (Garnworthy, 2004). We recently studied the influence of calving interval (CI, i.e. reproductive efficiency) on the CO_2_-eq footprint of lactating dairy cows using life cycle assessment methodology (Abreu et al., 2023). A comparison was made between production and CO_2_-eq/milk (corrected for fat and protein content) of cows with a CI of 13 or 15 months. The lactation period was estimated at 11 and 13 months for cows with a CI of 13 or 15 months, respectively (Cole and Null, 2009; Biassus et al., 2010). Total greenhouse gas emissions for 1 kg of milk (CO_2_-eq/milk) was 0.657 when the CI index was 13 months and 0.703 (7% increase) when the CI index was 15 months.

## Embryo technology to mitigate methane emission

Dairy cattle can suffer heat stress (HS) during summer which decreases dry matter intake (DMI), daily gain, milk yield, and fertility (Kadzere et al., 2002; Hansen, 2007). During HS, milk production decreases more than dry matter intake which increases the CO_2_-eq emission/kg energy-corrected milk (Rhoads et al., 2009). HS contributes to culling and death of cows (St-Pierre et al., 2003). The reduction in fertility is associated with altered ovarian folliculogenesis and oviductal function and increased embryonic mortality. The latter can be managed during periods of HS by replacing natural mating and artificial insemination (AI) with the transfer of either *in vivo* or *in vitro* derived embryos to cows on day 7 of the estrous cycle (Hansen, 2007; Baruselli et al., 2020).

**Figure 1:**
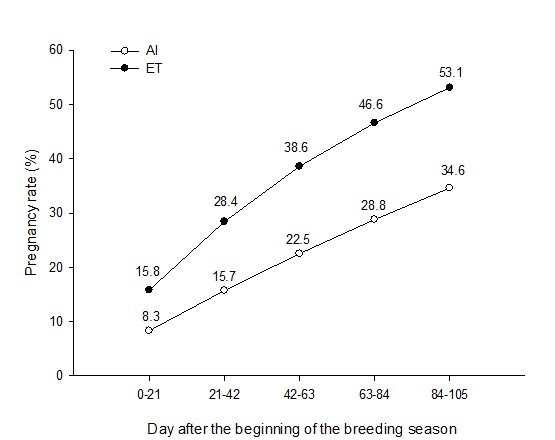
Survival curve assuming 60% service rate, 17% conception rate (P/AI) and 10% pregnancy rate every 21 days in repeat breeders and heat stressed dairy cows during 105-day AI program (pregnancy loss of 19% between 30 and 60 days gestation). For ET program, it was assumed 50% service rate (ET only in recipients with a corpus luteum), 40% conception rate (P/ET) and 15.3% pregnancy rate every 21 days in heat stressed dairy cows during a 105-day ET program (pregnancy loss of 21% between 30 and 60 days gestation). Adapted from Baruselli et al. (2018b).

We developed a simulation model which compared the use of AI or embryo transfer (ET) in HS dairy cows (Figure 1). The model assumed that pregnancy per AI (P/AI) and P/ET during HS were around 17 and 40%, respectively, and the service rate was 60% for AI and 50% for ET (ET was performed only on animals with a corpus luteum) (Baruselli et al., 2018b). The pregnancy rate following 105 days of breeding was 34.6% for AI and 53.1% for ET (53.6% increase). Cows subjected to AI had a greater number of days open (59.3 days) than cows exposed to ET (52.5 days) after the beginning of the breeding program. This shows that it is possible to increase the 21-day pregnancy rate by eight percentage points using ET in place of AI in HS dairy cows. As noted earlier, shorter inter-calving intervals are associated with a reduced CO_2_-eq budget in cattle.

As noted earlier in this review, the mature technologies of AI and MOET do not increase the rate of genetic gain. The latter is controlled by generation interval which is relatively long in cattle (Schefers and Weigel, 2012; Kasinathan et al., 2015). Generation interval can be shortened in cattle by utilizing oocytes from heifers early in life. Waves of follicular growth occur before birth and in the first weeks after birth in heifers (Evans et al., 1994a, 1994b; Monteiro et al., 2009). Oocytes can be recovered before birth (velogenesis; Betteridge et al., 1989; Georges and Massey, 1991; Kauffold et al., 2005) and well before puberty (Onuma et al., 1970; Baruselli et al., 2016), and used to generate viable embryos in the laboratory using *in vitro* embryo production (IVEP) (Baruselli et al., 2021). Prepubertal heifers show a good ovarian follicular response to FSH superstimulation and a relatively large number of oocytes can be retrieved for IVEP (Baruselli et al., 2021). IVEP is less efficient for oocytes from young heifers compared with mature heifers and cows and further research is needed to optimize IVEP in prepubertal heifers (Baruselli et al., 2021). Notwithstanding, IVEP with oocytes from young heifers has emerged as a fundamental enabling technology for the exploitation of genomic selection to produce cattle defined by efficiency, fertility and low CH_4_ emission.

## Balancing feed efficiency in meat and milk production with fertility and low CO_2_-eq emission

Cattle consume a relatively large amount of biomass and have a low feed conversion ratio compared with other livestock (FAO, 2018; Mottet et al., 2018). The provision of feed typically accounts for 70-80% of production costs in both extensive and intensive systems (Mottet et al., 2018). There is considerable interest, therefore, in identifying and multiplying cattle that have improved feed efficiency (Løvendahl et al., 2018; Davis and White, 2020). This applies to both extensive and intensive systems (Hietala and Juga, 2017; Kava et al., 2023). Associations between feed efficiency, methane production, and sustainability, have been known for more than 20 years (Arthur and Herd, 2005; Nkrumah et al., 2006; Freetly and Brown-Brandl, 2013). The relatively high heritability of growth and feed efficiency in cattle was recognised some 70 years ago and subsequently confirmed (Knapp and Nordskog, 1946; Berry and Crowley, 2013; Gonzalez-Recio et al., 2014; Sypniewski et al., 2021).

More recently, single nucleotide polymorphisms (SNPs) have been identified for feed efficiency in cattle and have been used in genomic selection (Arthur, 2015; Seabury et al., 2017; Sypniewski et al., 2021; Madilindi et al., 2022; Buss et al., 2023). As noted earlier, the relative abundance of ruminal acetogenic and methanogenic microbes influences methane emission by individual animals. There is a significant host effect on the ruminal microbe population, and it has been proposed that microbial gene abundance can be used to select cattle for feed efficiency and growth (Roehe et al., 2016). The genome of cattle can influence the population of ruminal microbes and hence the ruminal microbe genome profile which determines methane production (Difford et al., 2018; O’Hara et al., 2020; Gonzalez-Recio et al., 2023). Characterization of the ruminal microbe gene profile has been proposed as an alternative to expensive, time consuming methods for measuring feed efficiency in individual cattle (Arthur and Herd, 2005; Basarab et al., 2013; Kenny et al., 2018; Terry et al., 2021).

Growth and feed efficiency genes show single nucleotide polymorphism (Abo-Ismail et al., 2013; Seabury et al., 2017; Madilindi et al., 2022; Buss et al., 2023). Methane emission also shows single nucleotide polymorphism in cattle (Sarghale et al., 2020). The advent of molecular gene markers has created the opportunity to accurately identify cattle with desirable genes and to then use ART to rapidly multiply and disseminate cattle with improved feed efficiency and growth performance. Efficient cattle were reported in one study to have reduced CH_4_(g/day) and CO_2_-equivalent (g/day) emissions (Callegaro et al., 2022). The breeding technology used to generate efficient cattle will be governed by the production system and resources available. For example, AI and ET are already utilized in intensive dairy systems. Artificial insemination can be adopted in extensive beef systems as demonstrated in Latin America (Baruselli et al., 2004; Ferraz et al., 2012; Sartori et al., 2016; Mapletoft et al., 2018; Bó et al., 2018). Low-input, low-cost beef systems (North and South America, northern Australia, South Asia, Sub-Saharan Africa) will continue to rely on natural mating. For these regions, central breeding facilities will utilize genomic selection and ART to produce male embryos and/or bulls for dissemination for natural mating.

Whilst feed efficiency is undoubtedly a commercially important trait in beef and dairy cattle, selection for feed efficiency should not be at the expense of other important traits (Mu et al., 2016). As this review has argued, fertility has a major impact on enterprise productivity and profit in both beef and dairy systems. Studies in young growing British and European (*Bos taurus*) bulls consistently showed negative associations between feed efficiency and fertility measures including testicular growth and morphology and the characteristics of seminal plasma and spermatozoa (Awda et al., 2013; Fontoura et al., 2016; Montanholi et al., 2016; Bourgon et al., 2018). In contrast, a study in growing composite bulls (*Bos taurus* x *Bos indicus*) found that fertility measures did not differ for bulls of different feed efficiency (Kowalski et al., 2017).

Heifers with improved feed efficiency were reported to be leaner and reached puberty later than heifers with lesser feed efficiency (Randel and Welsh, 2013). In another study, heifers with good feed efficiency attained puberty earlier than heifers with poorer feed efficiency (Canal et al., 2020). Other studies in female cattle have also shown either a negative effect of feed efficiency on fertility (Mu et al., 2016; Ferreira et al., 2018) or no effect (Crowley et al., 2011; Davis et al., 2016). A study in dairy cows under commercial conditions reported that cows with high feed efficiency had a greater inter-calving interval (Vallimont et al., 2013). Dairy cattle selected for milk yield and feed efficiency had a reduced methane budget resulting from increased milk yield (Knapp et al. 2014). The impact of this selection strategy in an intensive dairy system was estimated to be a reduction of 9-19% in CO_2_-eq emission/kg energy-corrected milk (Knapp et al. 2014). In another study in dairy cows, selection based on genetic potential for milk production was associated with a decline in fertility, an increase in non-productive cows, and overall increase in CO_2_-eq emission for the production system (O’Brien et al., 2010). Another study in dairy cows reported low genetic correlations between methane production and fertility traits (Zetouni et al., 2018). Given the contrasting reports there is a need for further studies on feed efficiency, methane production, and lifetime fertility in cattle. The above studies have also demonstrated the importance of multi-trait selection in cattle breeding programs and the need to balance feed efficiency with other commercially important traits, in particular fertility (Bonamy et al., 2019).

## Enteric methane in production system life cycle assessment

Enteric methane forms part of the broader greenhouse gas (GHG) budget of beef and dairy production systems (Ibidhi and Calsamiglia, 2020). The broader GHG budget includes methane, nitrous oxide (N_2_O) and CO_2_ emission from manure, feed production, vehicles and transport, and other plant and equipment. The total GHG budget of a production system is determined by life cycle assessment (LCA) methodology standardized by ISO 14040 (ISO, 2006a) and ISO 14044 (ISO, 2006b) (de Vries et al., 2015; Kyttä et al., 2022). The relative contribution of different components of production systems to the GHG budget can vary greatly for different beef and dairy systems. One estimate for milk production was enteric methane 58.5% (CH_4_), feed production 29.4% (CO_2_, N_2_O) and manure 9.5% (CH_4_, N_2_O; FAO, 2018). The relative contribution of enteric methane can reach 91% in low efficiency systems (Chhabra et al., 2013). The digestibility of feed can also have a major impact on enteric methane contribution to the overall GHG budget (Pinares-Patiño et al., 2007; FAO, 2019; Eugéne et al., 2021; Congio et al., 2022). Herds with high fertility and high production efficiency have a reduced GHG budget (Strandén et al., 2022). In low fertility herds, replacement heifers can contribute up to 27% to the GHG budget (Garnworthy, 2004). The contribution of replacement heifers decreases to 10-12% in high fertility herds. High fertility herds with fewer replacement heifers require less feed production and have reduced manure, which lowers methane and nitrous oxide emission.

## Conclusions and future direction

The global attention on enteric CH_4_ production in cattle requires a response that involves collaboration between researchers and industry. Future generations of cattle will be characterized by better efficiency and fertility, which may reduce CH_4_ emission intensity. This will result from balanced multi-trait selection. There has been progress in the discovery of SNPs for efficiency and methane emission in cattle. These SNPs will be incorporated into assisted reproductive technology such as AI and ET for targeted multiplication and dispersal of cattle with defined production and environmental credentials. The urgency in moving to the next generation of cattle will see an increase in the production of embryos from genomically defined prepubertal heifers. This will reduce generation interval and accelerate the rate of genetic improvement to cattle defined by better efficiency and fertility and lower CH_4_ emission. The opportunity for cattle to be a part of ecosystem management was recently highlighted (Thompson et al., 2023). The challenge remains to communicate the importance of cattle for food security and the environment (Manzano et al., 2023).
